# Supplementary material for: Space- and time-resolved small angle X-ray scattering to probe assembly of silver nanocrystal superlattices
Source: Nat Commun. 2018 Oct 11;9:4211. doi: 10.1038/s41467-018-06734-9 (PMC6181943; doi:10.1038/s41467-018-06734-9)
Supplement: Supplementary file 1 — Supplementary Information [file 41467_2018_6734_MOESM1_ESM.pdf]

Supplementary Information

Space- and Time-Resolved Small Angle X-ray Scattering to Probe Assembly of Silver Nanocrystal Superlattices

Yu et al.

Space- and Time-Resolved Small Angle X-ray Scattering to Probe Assembly of Silver Nanocrystal Superlattices

Yixuan Yu[1], Dian Yu[2], Babak Sadigh[1] and Christine A. Orme[1*]

[1] Lawrence Livermore National Laboratory, 7000 East Avenue, Livermore, CA 94550, USA.

[2] University of California, Los Angeles, 410 Westwood Plaza, Los Angeles, CA 90095, USA.

[*] email: orme1@llnl.gov

Contents

Supplementary Figures

Supplementary Figure 1: Additional GISAXS patterns of superlattices

Supplementary Figure 2: Additional atomic force microscopy data

Supplementary Figure 3: Additional optical microscopy images

Supplementary Figure 4: Water fall plots of SAXS data for assembly under an applied field strength of 14 Vcm^{-1}

Supplementary Figure 5: Water fall plots of SAXS data for assembly under an applied field strength of 28 Vcm^{-1}

Supplementary Figure 6: Water fall plots of SAXS data for assembly under an applied field strength of 57 Vcm^{-1}

Supplementary Figure 7: Water fall plots of SAXS data for assembly under an applied field strength of 86 Vcm^{-1}

Supplementary Figure 8: Size distribution of the original nanocrystal solution

Supplementary Figure 9: Kratky plots of nanocrystal solution under 57 Vcm^{-1} electric field for 0 and 25 min.

Supplementary Figure 10: Additional contour plots of nanocrystal solution volume fraction against the distance to the anode and the duration of the electric field

Supplementary Methods

1. Design and layout of in situ small angle X-ray scattering cell

2. Scanning electron microscopy and atomic force microscopy
3. Fitting of form factors
4. Concentration-to-volume fraction conversion
5. Calculating the nanocrystal flux and velocity from volume fraction profiles

Supplementary Notes

Supplementary Note 1: Surface versus solution superlattice nucleation

Supplementary Note 2: Merging of superlattices

Supplementary Note 3: Estimating the nucleation density of superlattices

Supplementary References

Supplementary Figures

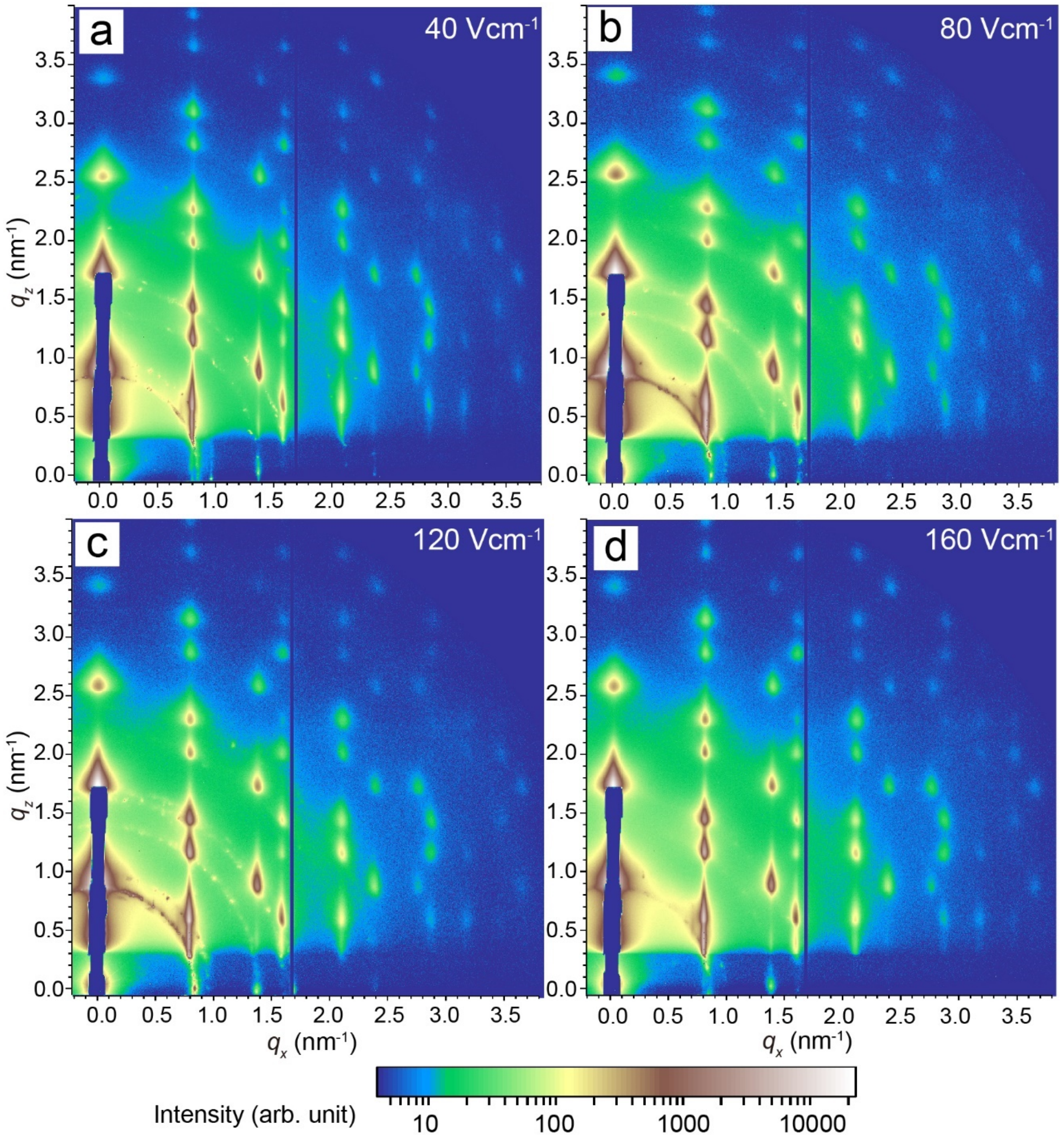

Supplementary Figure 1. GISAXS patterns of superlattices made under applied field strengths of (a) 40 Vcm^{-1}, (b) 80 Vcm^{-1}, (c) 120 Vcm^{-1}, and (d) 160 Vcm^{-1} for 60, 30, 15, and 9 min, respectively. GISAXS patterns can be indexed to highly ordered face centered cubic structures. The axes, q_x and q_z represent the scatter vector in the plane of the sample and perpendicular to the plane of the sample, respectively.

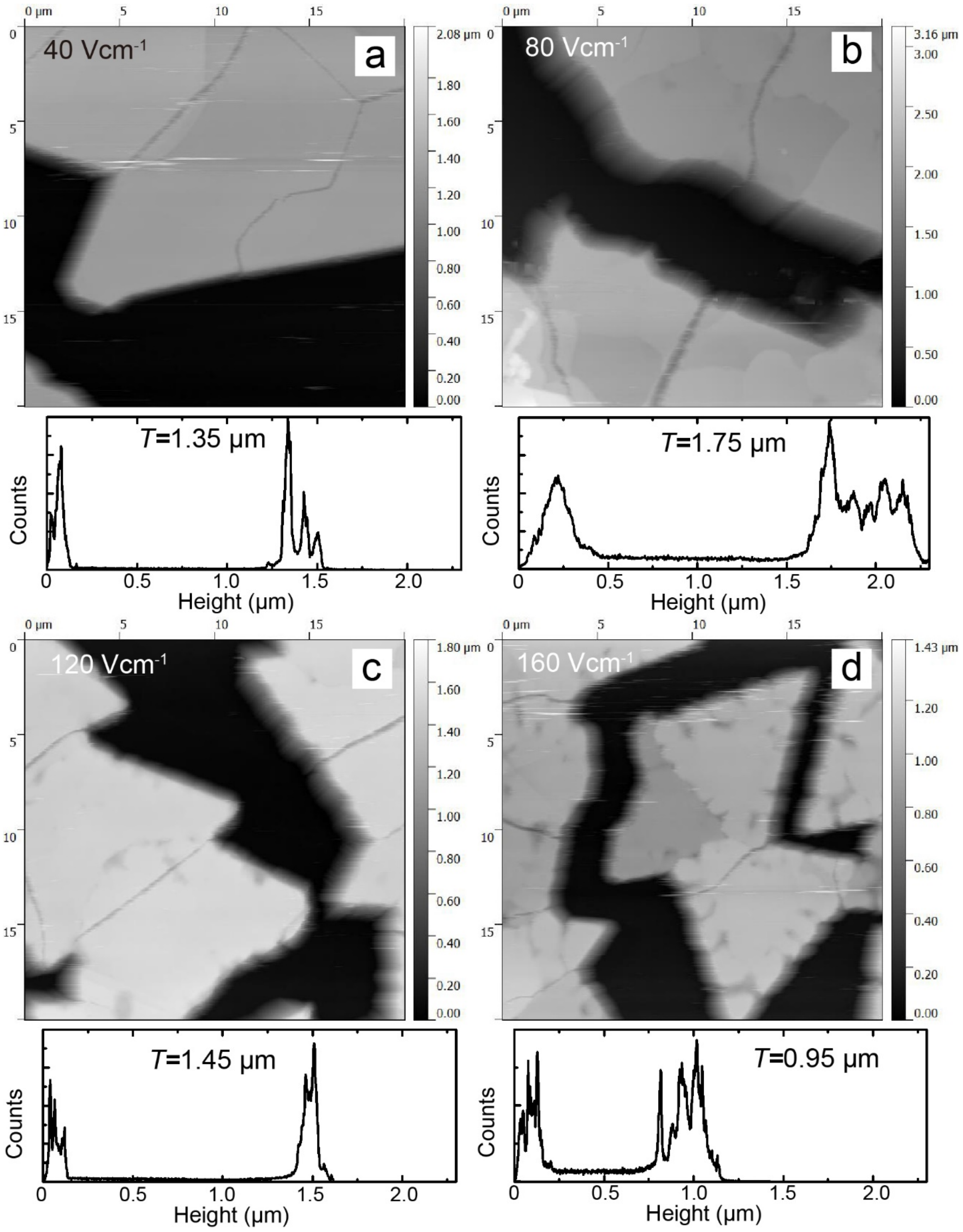

Supplementary Figure 2. AFM images and the extracted height profile for superlattices grown under applied field strengths of (a) 40 Vcm^{-1}, (b) 80 Vcm^{-1}, (c) 120 Vcm^{-1}, and (d) 160 Vcm^{-1} for 60, 30, 15, and 9 min, respectively. *T* represents the film thickness.

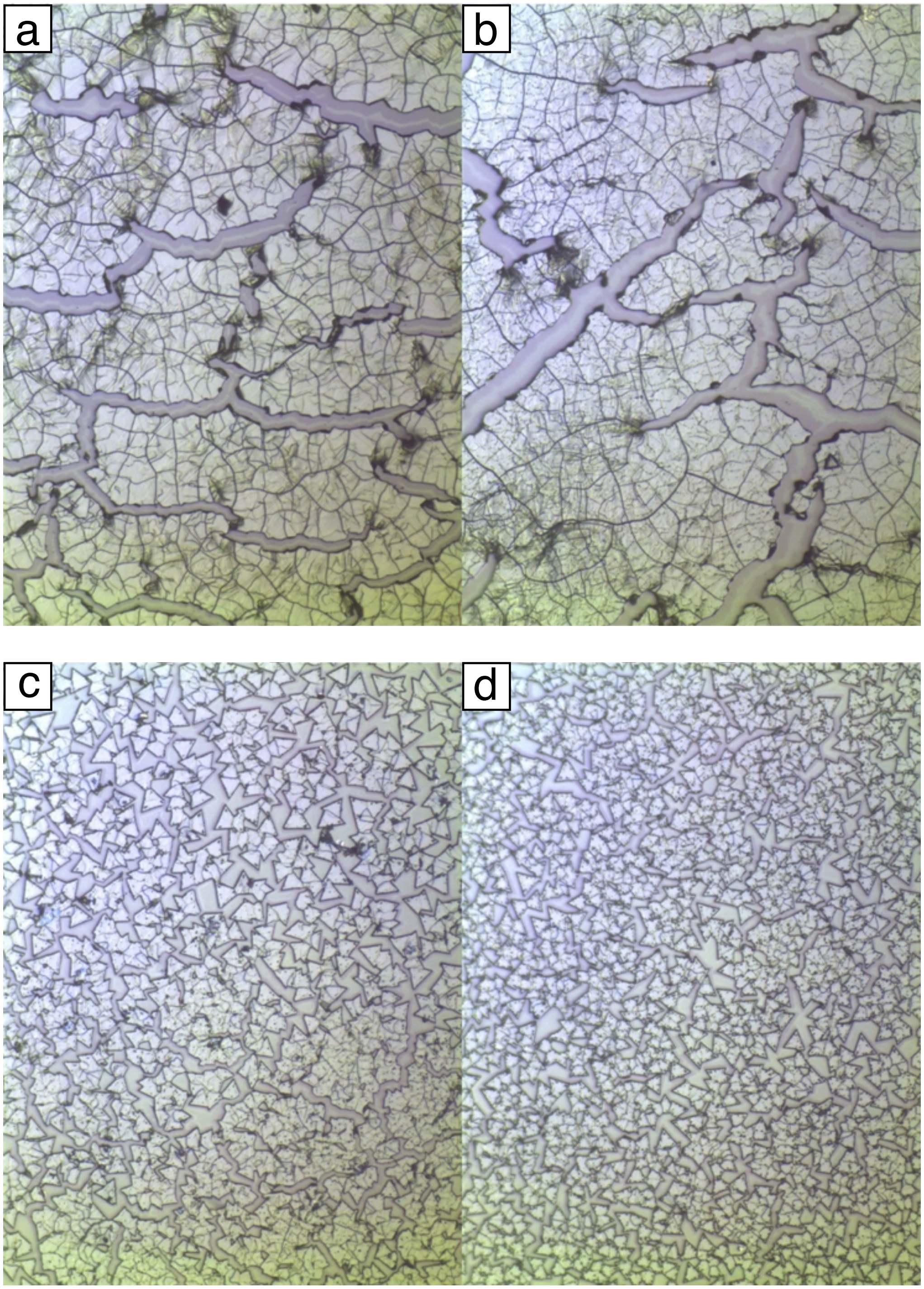

Supplementary Figure 3. Optical microscopic images of superlattices grown under applied field strengths of (a) 40 Vcm^{-1}, (b) 80 Vcm^{-1}, (c) 120 Vcm^{-1}, and (d) 160 Vcm^{-1} for 60, 30, 15 and 9 minutes with superlattice coverage of approximately 85, 85, 70, and 75%, respectively. Error bars for the coverage are estimated to be 10% based on uncertainty in defining the film edges.

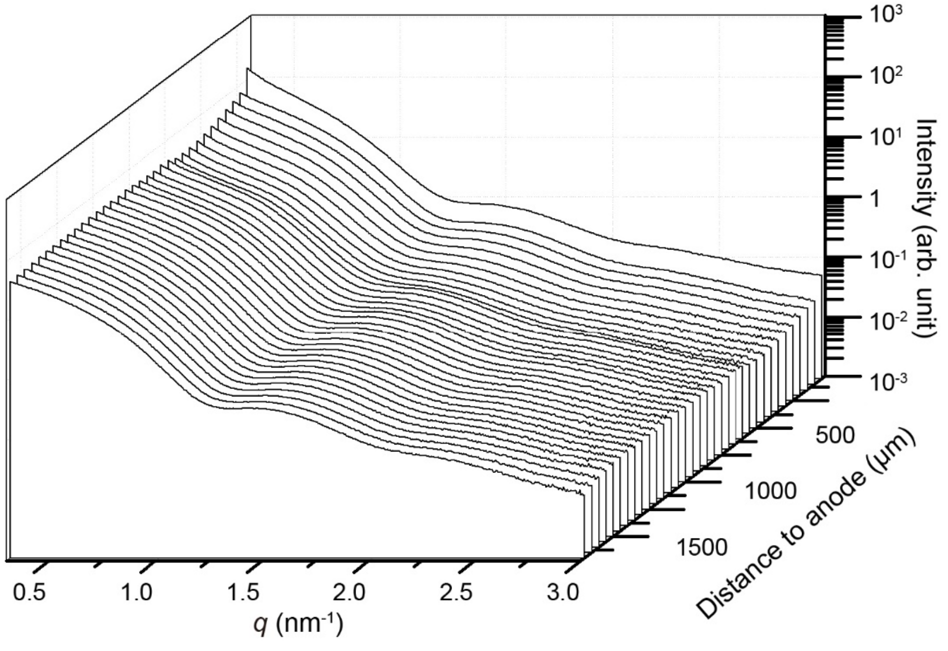

Supplementary Figure 4. Water fall plot of SAXS data for the system under an applied field strength of 14 Vcm^{-1}electric field for 155 min.

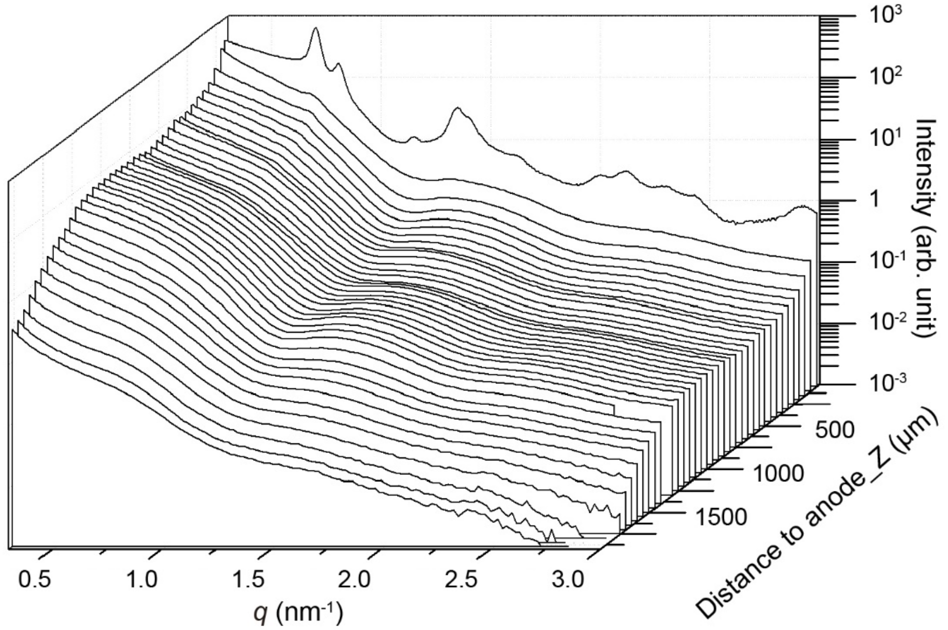

Supplementary Figure 5. Water fall plot of SAXS data for the system under an applied field strength of 28 Vcm^{-1} for 95 min.

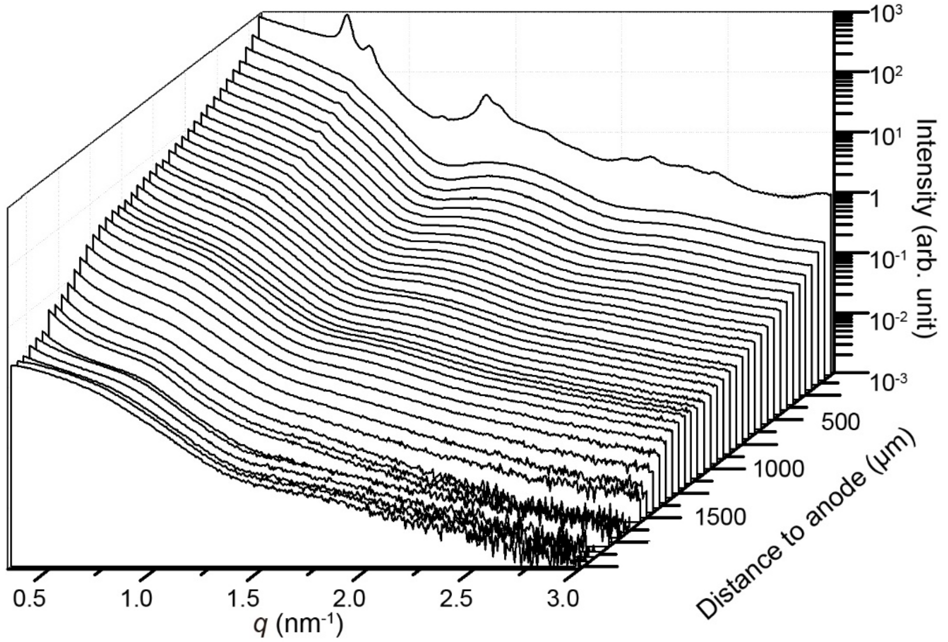

Supplementary Figure 6. Water fall plot of SAXS data for the system under an applied field strength of 57 Vcm^{-1} for 55 min.

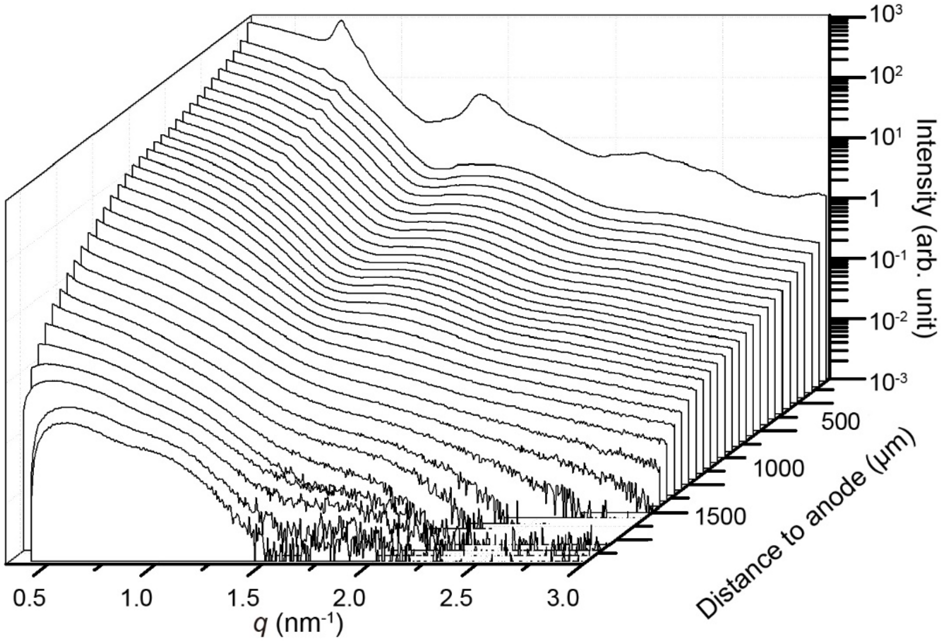

Supplementary Figure 7. Water fall plot of SAXS data for the system under an applied field strength of 86 Vcm^{-1} for 35 min.

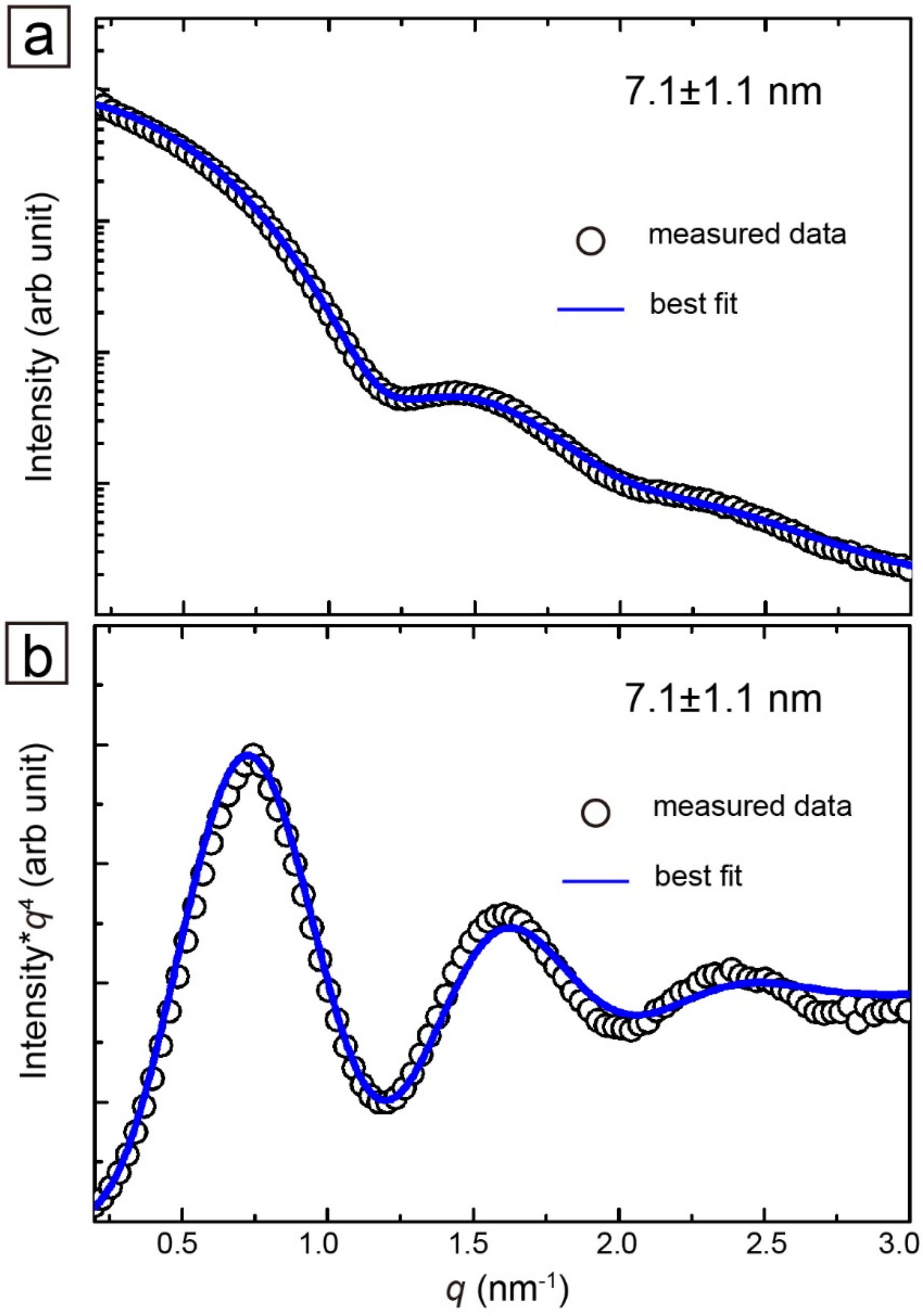

Supplementary Figure 8. Solution SAXS data (black circles) of the nanocrystal solution prior to applying an electric field, fitted (blue solid line) with form factors of a collection of solid spheres of a Gaussian size distribution as described in Supplementary Method 3. (a) The top plot is radially integrated intensity plot, (b) the bottom is the Porod plot. The abscissa, *q,* represents the magnitude of the scatter vector. Fits correspond to an average size of 7.1 nm and standard deviation of 1.1 nm.

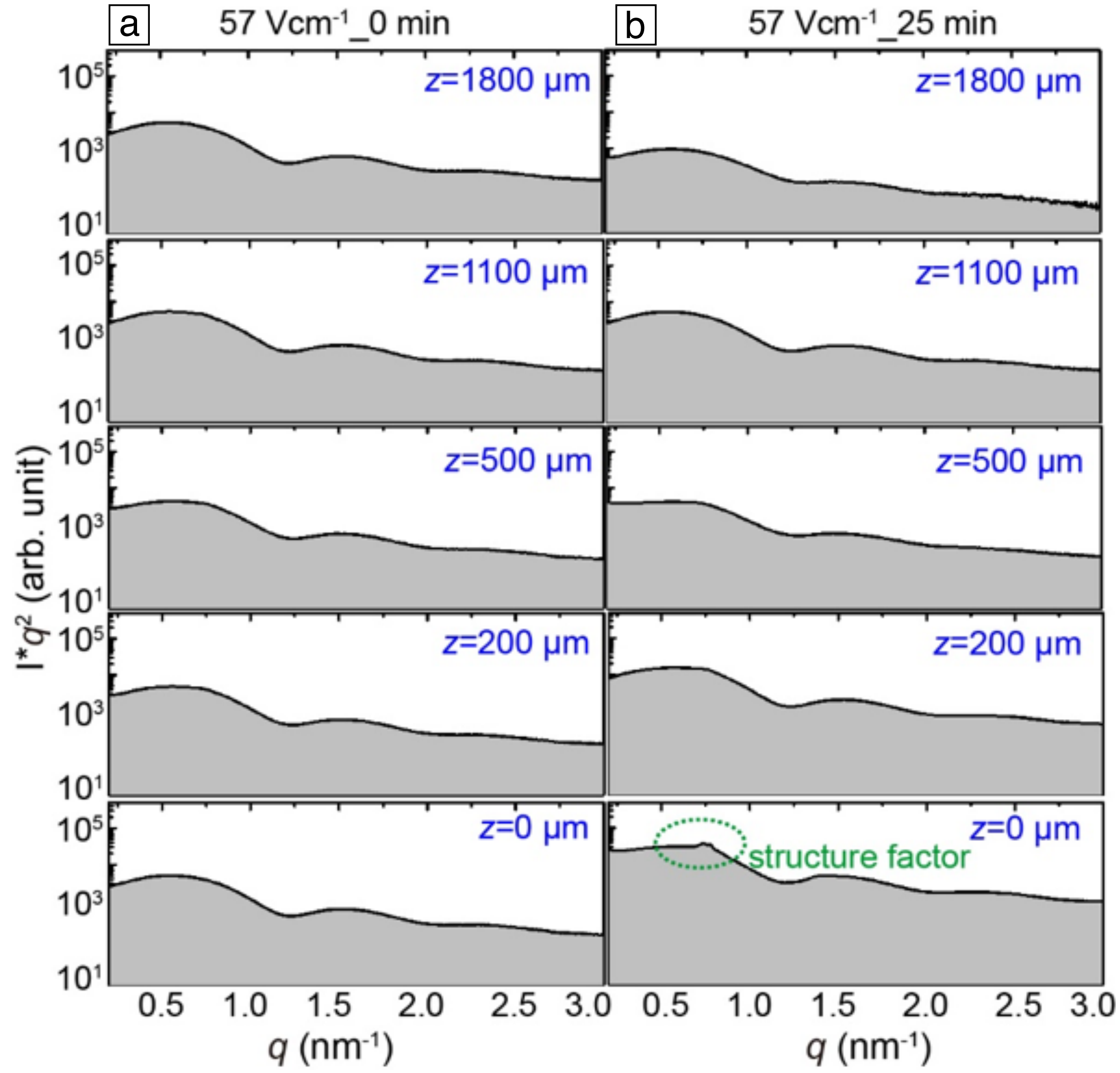

Supplementary Figure 9. Kratky plots from SAXS data of nanocrystal solution at various distances to the anode, prior to (a) and after applying 57 Vcm^{-1} electric field for 25 min (b). In these plots *I* represents the intensity, *q*, the magnitude of the scatter vector, and *z*, the distance from the anode.

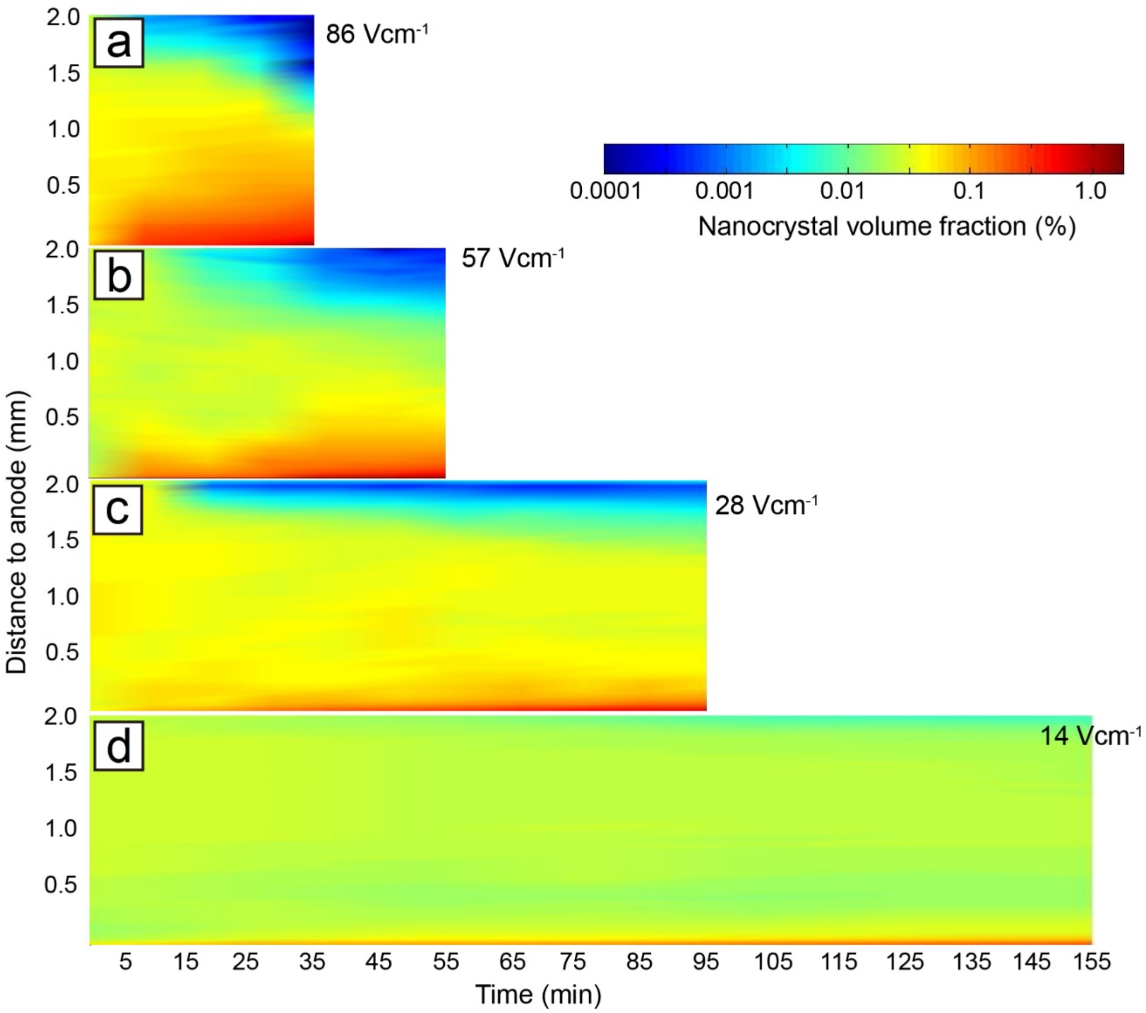

Supplementary Figure 10. Contour plot of nanocrystal solution volume fraction against distance to the anode and time of applying electric field for *in situ* experiments performed with applied electric field strengths of (a) 86 Vcm^{-1}, (b) 57 Vcm^{-1}, (c) 28 Vcm^{-1}, and (d) 14 Vcm^{-1}.

Supplementary Methods

Design and layout of *in situ* small angle X-ray scattering (SAXS) cell

In situ SAXS is performed at the Advanced Photon Source (APS) beamline 12-ID-B with finely focused 13.3 keV radiation that has a full width at half maximum (FWHM) of 34 μm. As shown in Supplementary Fig. 11, the nanocrystal solution is loaded into the liquid chamber and electric field is generated between the top (cathode) and bottom electrodes (anode). The distance between the electrodes is 3.5 mm and the thickness of the liquid chamber, which is also the path length of X-ray through the solution, is 10 mm. Nanocrystal solution is injected through a pin hole on the top of the cell, which is sealed with a piece of tape during the experiment. The X-ray beam is aligned to be parallel to the anode and scanned through the sample from the anode to bulk solution at a step size of 50 μm for a total of 40 steps. The exposure time at each step of the scan is 10 sec. The cell is shifted horizontally by 100 μm between each scan to reduce the beam damage.

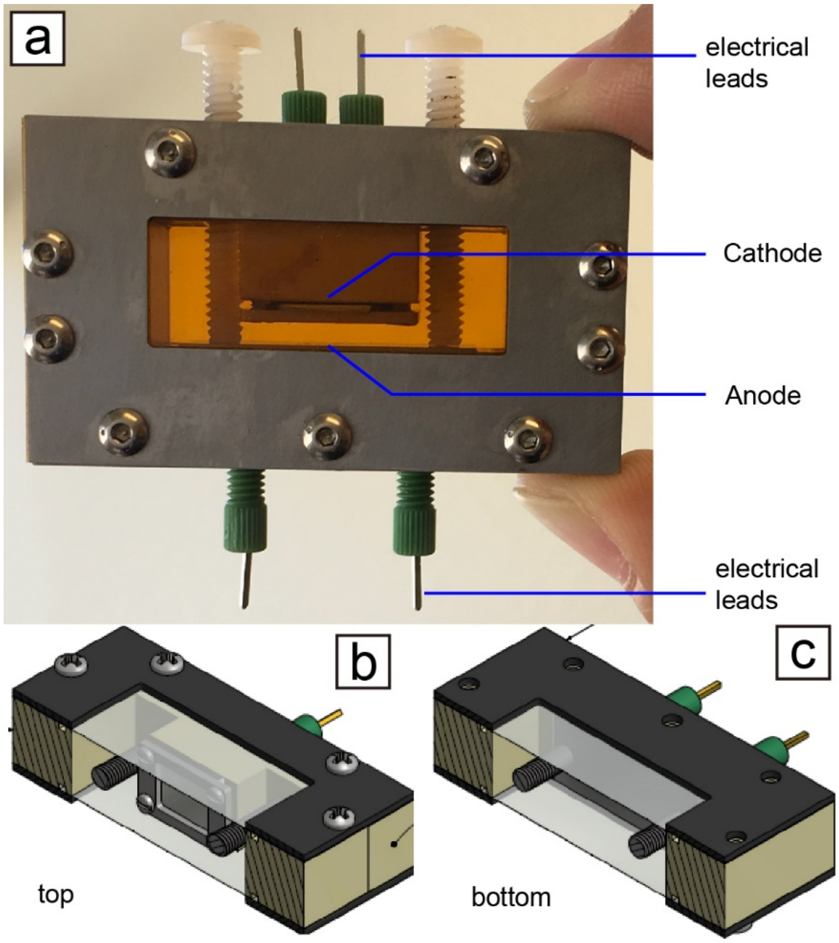

Supplementary Figure 11. (a) Picture of the custom *in situ* SAXS cell with drawings of the (b) top and (c) bottom halves of the cell.

Scanning electron microscopy (SEM) and atomic force microscopy (AFM)

SEM was performed with a FEI Nova 600 DualBeam SEM/FIB system operated at 10 kV accelerating voltage, using an In-lens SE detector. AFM is performed with an Asylum Research MFP-3D AFM using contact mode with a TR800 PSA silicon nitride tip. AFM data were analyzed with Gwyddion software (available for download from http://gwyddion.net).

Fitting of form factors

The scattering intensity expected for a collection of non-interacting solid spheres can be calculated as[1,2]

$$I(q) \propto \int_0^\infty N(R)P(qR)R^6 dR \tag{1}$$

in which q is the scatter vector, $N(R)$ is the nanocrystal size distribution, which is assumed to be Gaussian with average radius $\bar{R}$ and standard deviation σ:

$$N(R) = \frac{1}{\sigma\sqrt{2\pi}} \exp\left[\frac{-(R-\bar{R})^2}{2\sigma^2}\right] \tag{2}$$

and form factors $P(qR)$ for homogenous spheres is:

$$P(qR) = \left[3\,\frac{\sin(qR) - qR\cos(qR)}{(qR)^3}\right]^2 \tag{3}$$

Combining Supplementary Equations 1-3 allows us to fit the scattering data of nanocrystal solutions and find the average diameter $\bar{d} = 2\bar{R}$, standard deviation $\delta = 2\sigma$, and size polydispersity ${}^{\delta}/_{\bar{d}}$.

Concentration-to-volume fraction conversion

The nanocrystal solution concentration (c) with units of mg/mL can be converted to volume fraction of Ag cores (ϕ_{core}) by

$$\phi_{\text{core}} = {}^{c}/_{\rho} \tag{4}$$

in which ρ is the density of bulk Ag, assuming the mass of ligands are negligible. The volume of Ag nanocrystals consists of both cores and capping ligands. The volume fraction of Ag nanocrystal is therefore

$$\phi = \phi_{\text{core}} \left(\frac{d+2l}{d}\right)^3 \tag{5}$$

in which d is the core diameter and l is the effective ligand length.[3] Core diameter can be estimated with the average nanocrystal diameter obtained by fitting the solution SAXS data.

Effective ligand length can be calculated from the lattice constant (a) determined by GISAXS from

$$l = \frac{1}{2}(\frac{a}{\sqrt{2}} - d) \tag{6}$$

Calculating the nanocrystal flux and velocity from volume fraction profiles

The continuity equation[4] can be used to relate the nanocrystal flux (J) and the number density (c),

$$\frac{\partial J(z,t)}{\partial z} + \frac{\partial c(z,t)}{\partial t} = 0 \tag{7}$$

where the number density c is related to the volume fraction Φ by $c = {}^{\Phi}/_{V_{\mathrm{NC}}}$, and V_{NC} is the effective volume of nanocrystal. By integrating over space, Supplementary Equation. 7 can be rewritten as:

$$J(z,t) - J(0,t) = -\frac{\partial(\int_0^z c\,(z,t)dz')}{\partial t} \tag{8}$$

Given the boundary condition that nanocrystals cannot penetrate the electrodes, $J(0,t) = 0$. The flux can be expressed as:

$$J(z,t) = -\frac{z}{V_{\mathrm{NC}}}\frac{\partial[\overline{\Phi(z,t)}]}{\partial t} \tag{9}$$

where $\overline{\Phi(z,t)} = \frac{1}{N}\sum_1^N \Phi[(N-1)\Delta z, t]\,, z = N\Delta z, \Delta z = 50$ µm is the nanocrystal volume fraction spatially averaged over the area from the anode to the position z and the negative sign is due to that the flux is toward anode, which is the opposite to direction of increasing z. For example, in order to calculate the flux at $z = 50$ µm, we need to know the time dependence of $\overline{\Phi(50\mu\mathrm{m}, t)} = \Phi(0\ \mu\mathrm{m}, t)$, which is equal to the slope of the linear fits in Figure 4b in the main text.

At any position (z) within the solution, the nanocrystal flux, $J(z,t)$, is given by the drift-diffusion equation with contributions from the electric field driven nanocrystal flow towards the anode and the diffusion driven flow away from the anode,

$$J(z,t) = c(z,t)v(z) - D\frac{\partial c(z,t)}{\partial \mathrm{z}} \tag{10}$$

in which v and D are nanocrystal velocity and diffusion coefficient, respectively. The velocity is related to the local electric field by $v(\mathrm{z}) = \mu E(\mathrm{z})$, where µis the electric mobility and we expect the local field strength, $E(z)$, to scale with the applied electric field. As shown in Figure 4a in the main text, at the point $z = 800$ µm, near the middle of the electrodeposition chamber, the concentration gradient become approximately negligible,

$\left.\frac{\partial\Phi(z,t)}{\partial z}\right|_{z=800\ \mu m} \to 0$, and the right part of equation is reduced to the first term. This simplification allows us to calculate the nanocrystal velocity from the flux and the measured concentrations.

Supplementary Fig. 12a plots the average volume fraction for the solution between $z = 0$ µm and $z = 800$ µm, $\overline{\Phi(800\mu m, t)}$, against time for various field strength. At $z = 800$ µm, the nanocrystal volume fraction is approximately independent of time, allowing us to treat $c(800\mu m, t)$ as a constant. The absolute value of the nanocrystal velocity is calculated and plotted in Supplementary Fig. 12b, with a unit of µm s^{-1}. The electric field-driven drift velocity of nanocrystals is a function of the applied electric field strength, as expected.

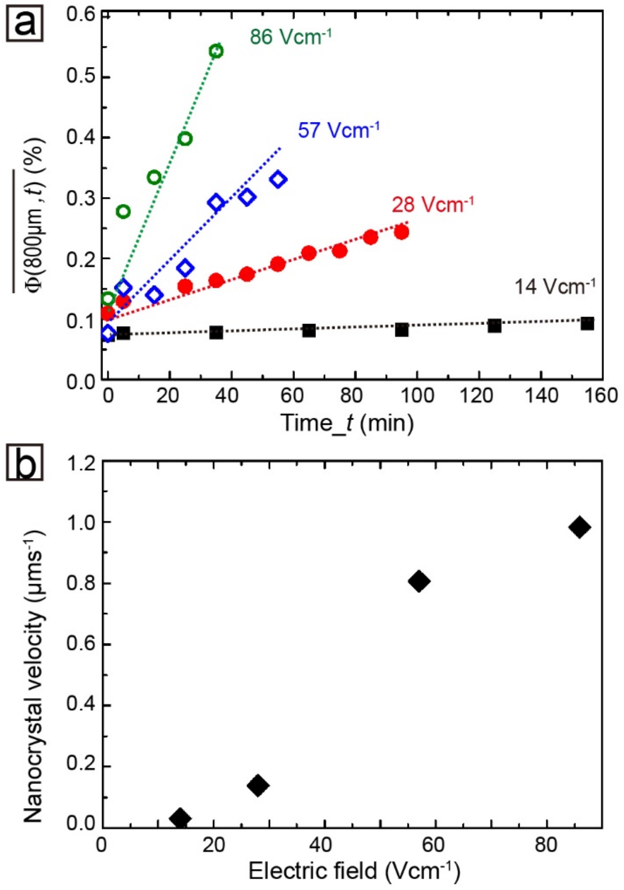

Supplementary Figure 12. (a) Nanocrystal volume fraction (Φ) versus time, spatially averaged between the anode and a position 800 µm away, under various electric field strengths. Black squares, red solid circles, blue open diamonds, and green open circles represent fields of 14, 28, 56, and 86 Vcm^{-1}, respectively. The dotted lines are linear fits. (b) Calculated drift velocity of nanocrystals 800 µm away from the anode as a function of applied electric field strength.

Supplementary Notes

Supplementary Note 1: Surface versus solution superlattice nucleation

Most of the superlattices are nucleated on the surface of substrate. These superlattices are oriented on the substrate with their (111) planes parallel to the surface, which is the majority orientation as shown by GISAXS. The superlattices highlighted with red circles in Supplementary Fig. 13 have likely nucleated in the solution and subsequently land on the substrate during drying because they have a octahedral shape with a random orientation relative to the substrate.

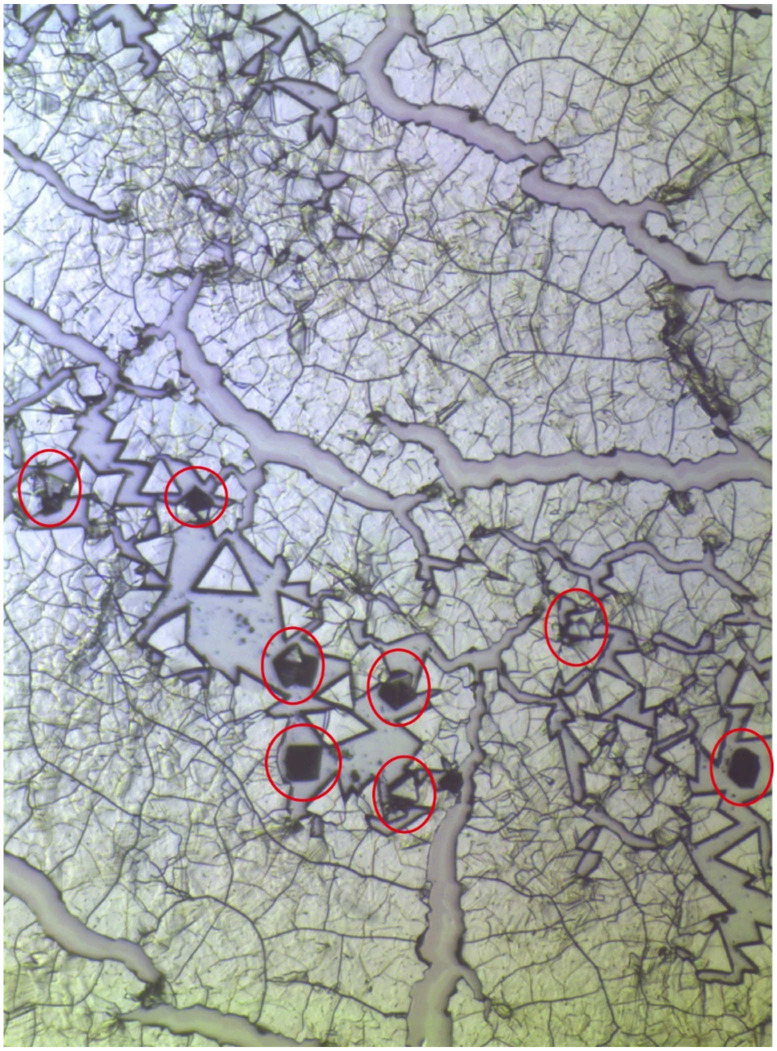

Supplementary Figure 13. Optical microscope image showing that most of the superlattices are nucleated on substrate surface while superlattices nucleated in the solution, highlighted with red circles, can also be observed.

Supplementary Note 2 - Merging of superlattices

Superlattices nucleate on the anode and then grow. If two nuclei are close to one another, the superlattices may grow into one another and merge into one. Supplementary Fig. 14a shows a SEM image for merged superlattices, and the zoomed-in image of the area highlighted with the white dashed rectangle is show in Supplementary Fig. 14b. Boundaries of superlattices are clearly visible. The growth edges, reflecting the orientation of nanocrystal packing in the superlattices, are also visible. Images (Supplementary Fig. 14c and 14d) that are further zoomed in to the area highlighted with green and blue dashed rectangles reveal that superlattices grains have slightly different heights, causing the grain boundaries to be visible in SEM. Besides the contrast caused the by height differences, grain boundaries can also be identified by the discontinuity of grow edges. This is because the superlattices nucleate independently, and the merged superlattices often have different orientations, leading to the bending of growth edges at the boundary.

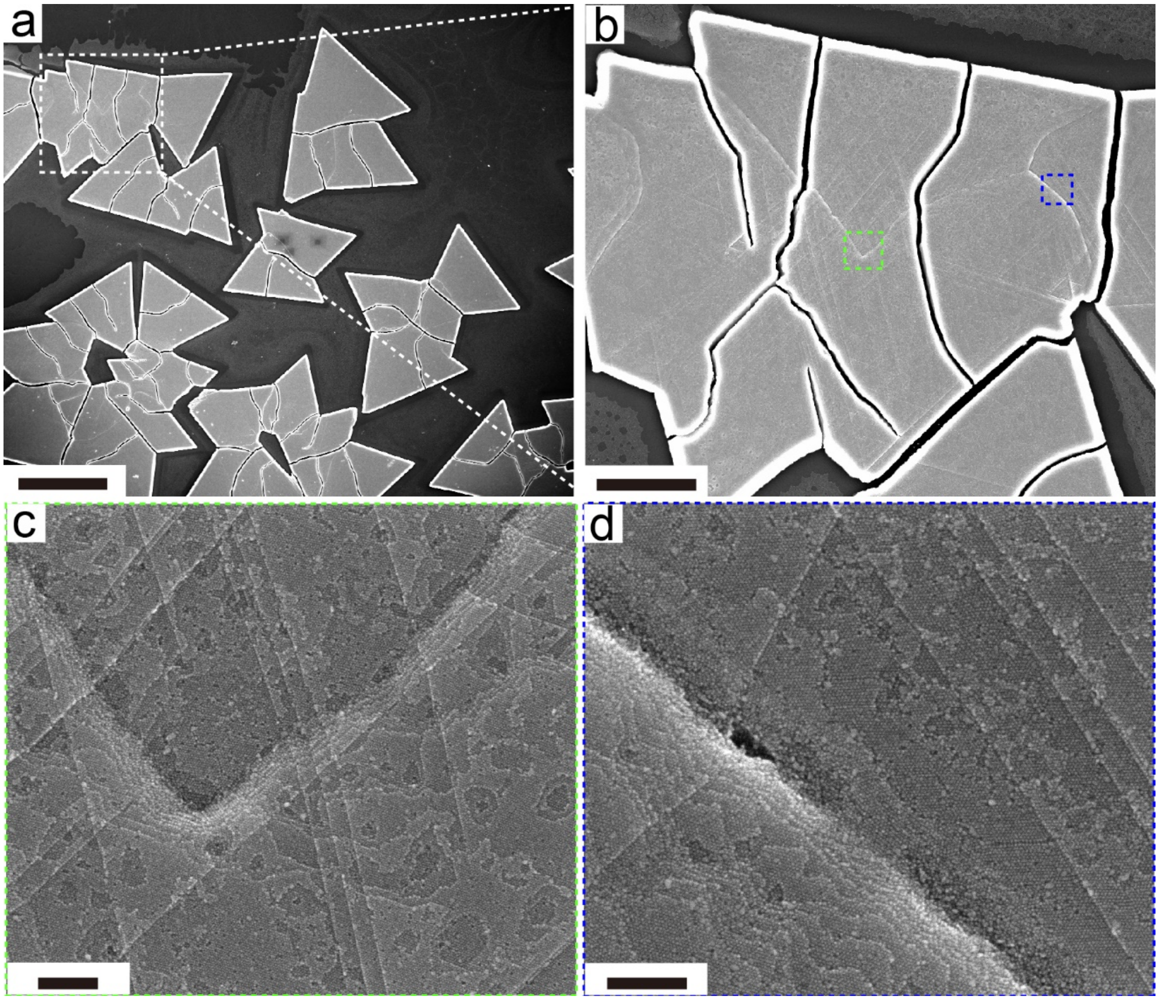

Supplementary Figure 14. (a) SEM image of merged superlattices grown under an applied field strength of 20 Vcm^{-1}. Scale bar is 20 µm. (b) SEM image zoomed in for the

area highlighted with white dashed rectangle in (a), showing grain boundaries and growth edges. Scale bar is 5μm. (c) and (d) SEM images zoomed in for the areas highlighted with green and blue dashed rectangles in (b) with 200nm scale bars. Superlattice grains are slightly different in height and have different nanocrystal packing orientations.

Merged superlattices continue to grow, and merge with others, eventually leading to the formation of films, as shown in the Supplementary Fig. 15.

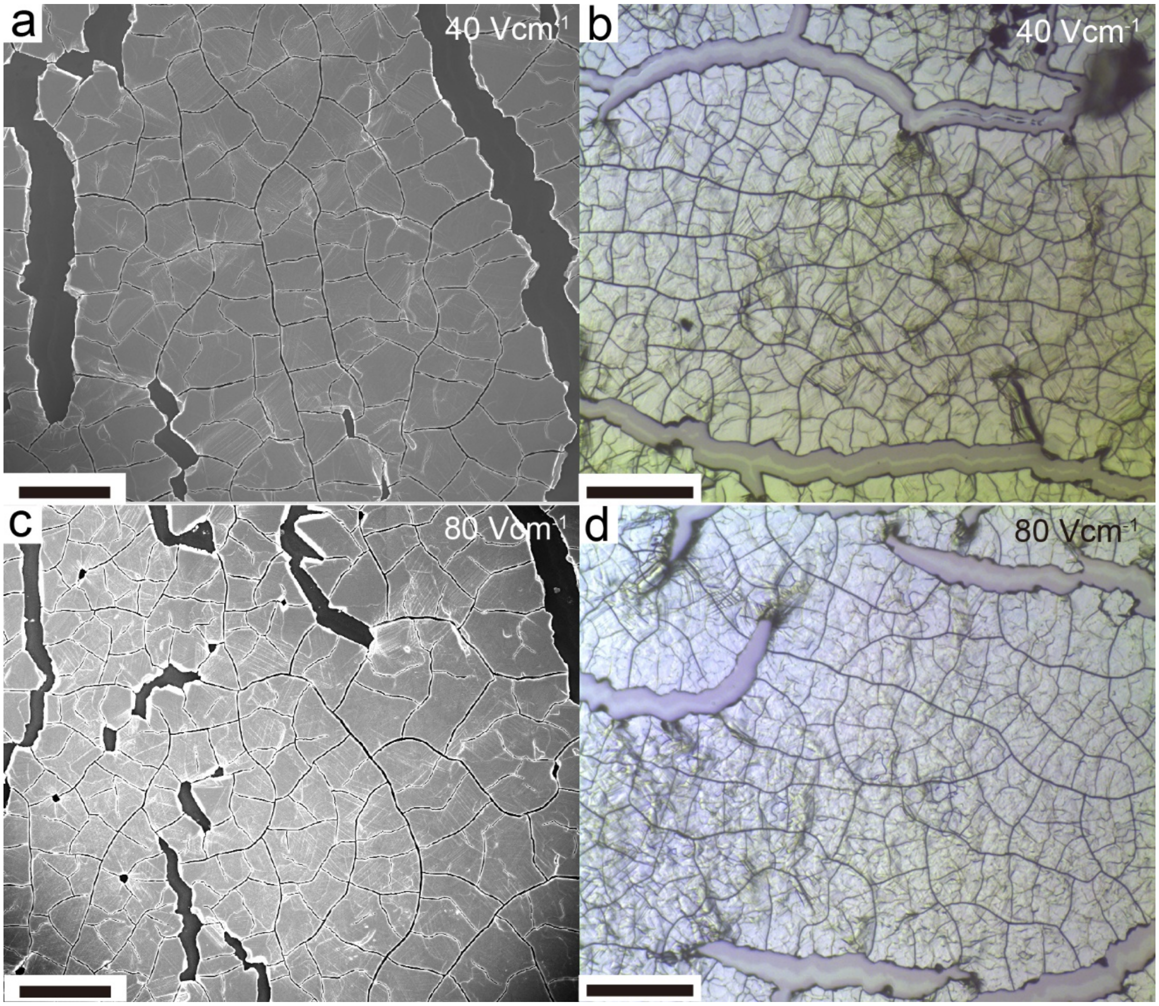

Supplementary Figure 15. (a) SEM image with 20μm scale bar and (b) optical microscopic image with 50 μm scale bar showing superlattices made under 40 Vcm^{-1}for 60 min that have merged into a film. (c) SEM image with 20μm scale bar and (d) optical microscopic image with 50 μm scale bar showing superlattices made under 80 Vcm^{-1}for 30 min that have merged into a film.

Supplementary Note 3 - Estimating the nucleation density of superlattices

The nucleation density is estimated based on the assumption that each nucleus grows into a superlattices. Then the nucleation density becomes the number density of superlattices on the anode, which can be directly counted. Counting the number of superlattices is straight forward if they are not or only slightly merged, as shown in Figure 2 in the main text. When superlattices are completely merged into a film, we need to identify the grain boundaries before counting the number of superlattices. As discussed in the Supplementary Note 1, we can identify the grain boundaries by (1) the contrast in SEM images, and (2) discontinuities of grow edges. Supplementary Fig. 16 further illustrates these ideas.

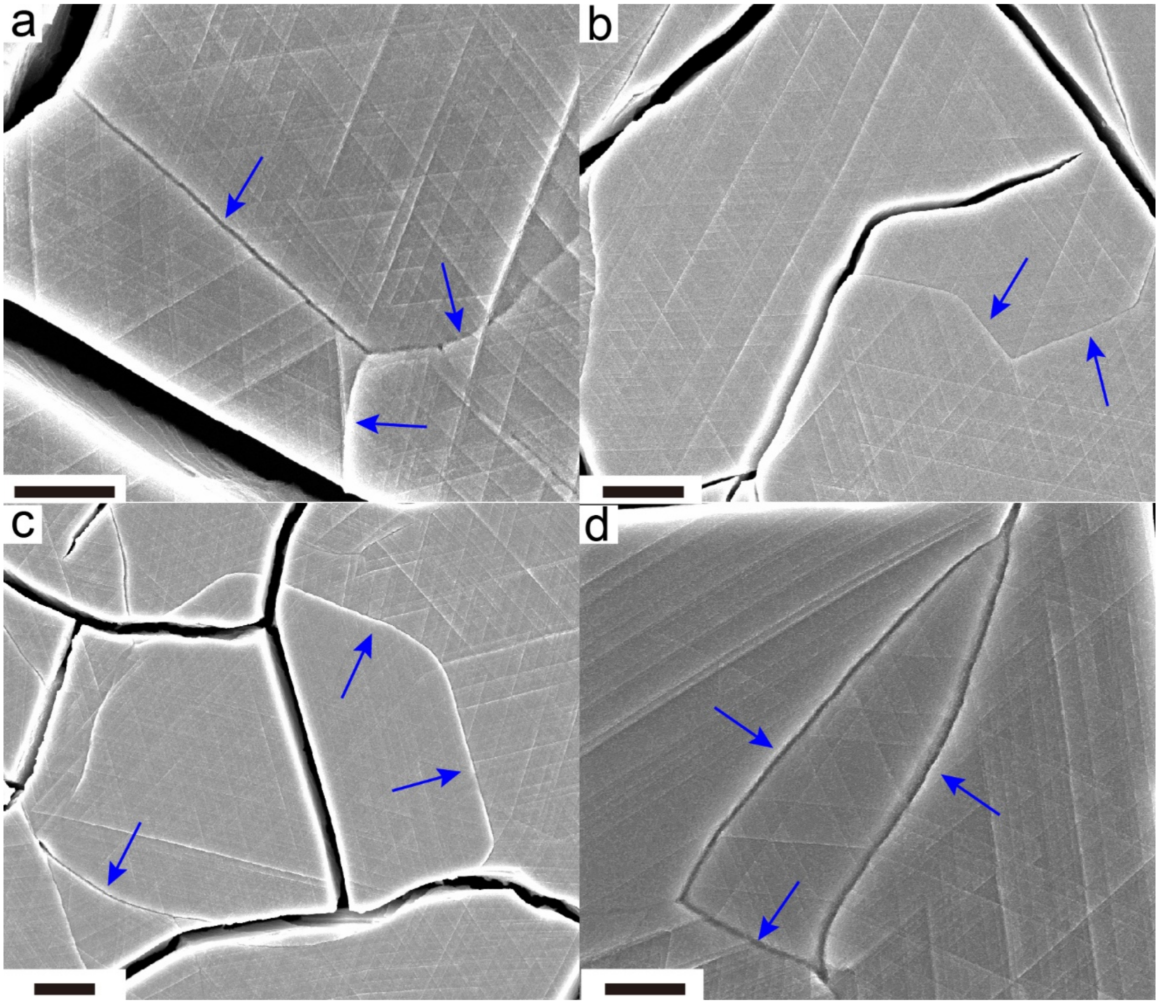

Supplementary Figure 16. SEM images showing grain boundaries, highlighted with blue arrows, of merged superlattices. (a) and (d) have 1 μm scale bars and (b) and (c) have 2 μm scale bars.

Supplementary Fig. 17 shows examples of superlattice number counting with SEM images. We first identify the grain boundaries and highlight them with blue dashed lines, and then mark superlattices grains one-by-one with red numbers. For superlattices grains partially present in the image, we estimate their area and count them as one when their combined area is close to the average grain area. The number density of superlattice grains, assumed to be equal to the nucleation density, is the ratio between the number of superlattice grains in the image and the area covered by the image.

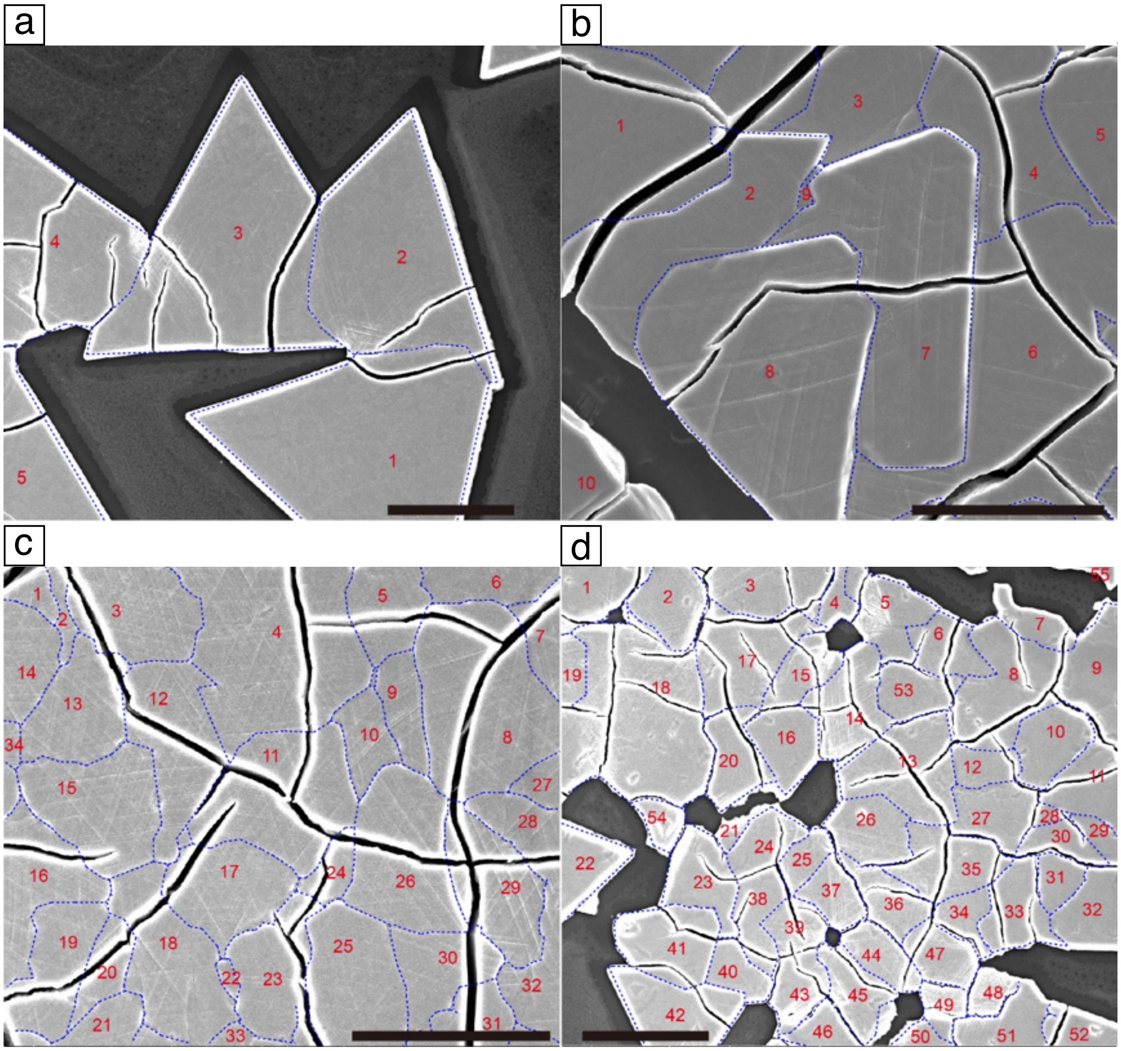

Supplementary Figure 17. Examples of counting the number of superlattices from SEM images of films deposited with applied electric fields of (a) 20 Vcm^{-1}, (b) 40 Vcm^{-1}, (c) 80 Vcm^{-1}, and (d) 120 Vcm^{-1}. All images have 10 μm scale bars.

Supplementary References

(1) Li, T.; Senesi, A. J. & Lee, B. Small Angle X-ray Scattering for Nanoparticle Research. *Chem. Rev.* **116**, 11128-11180 (2016).

(2) Yu, Y. et al. Cooling Dodecanethiol-Capped 2 nm Gold Nanocrystal Superlattices below Room Temperature Induces a Reversible Order-Disorder Structure Transition. *J. Phys. Chem. C.* **120**, 27682-27687 (2016).

(3) Korgel, B. A., Fullam, S., Connolly, S. & Fitzmaurice, D. Assembly and Self-Organization of Silver Nanocrystal Superlattices: Ordered "Soft Sphere". *J. Phys. Chem. B* **102**, 8379-8388 (1998).

(4) Cushman-Roisin, B. & Beckers, J.-M. *Introduction to Geophysical Fluid Dynamics*. (Academic Press, London, 2009).
